# Persons’ experiences of having hypertension: An interview study

**DOI:** 10.1016/j.ijnsa.2022.100071

**Published:** 2022-02-19

**Authors:** Assistant Senior Lecturer Helena Rosén, Associate Professor Eva I Persson, Lecturer Rebecca Gagnemo Persson, Associate Professor Eva Drevenhorn

**Affiliations:** Department of Health Sciences, Faculty of Medicine, Lund University, Box 157, Lund, 221 00 Sweden

**Keywords:** Hypertension, Interview, Patient perspective, Self-care

## Abstract

**Background:**

among the 1–1.5 billion persons with hypertension globally only, 20–30% have controlled blood pressure (BP). The most important problem identified is non-adherence to treatment, i.e., failure to change lifestyle and to take prescribed medication. Knowledge about the reasons for this is limited.

**Objectives:**

The aim of the study was to explore people's experiences of having hypertension.

**Design:**

Inductive design based on qualitative interviews.

**Settings:**

The south of Sweden.

**Participants:**

Twelve adults diagnosed with hypertension and treated in primary care were interviewed.

**Methods:**

The transcribed interviews were analysed using content analysis, which rendered three categories.

**Results:**

The individuals adapted to their diagnosis in different ways. Collaboration with the staff gave security, but the persons still perceived anxiety and uncertainty.

**Conclusions:**

To meet the needs of people with hypertension, strategies such as person-centred counselling and care, using digital interventions, following national guidelines and starting nurse-led clinics, may be of help. These strategies can give a foundation for increased self-efficacy, which is crucial for persons to be able to change lifestyle and adhere to prescribed medication in order to achieve BP control.

## 1 Introduction

Worldwide there are 1–1.5 billion (WHO, 2015) persons diagnosed with hypertension. In Sweden 2.5 million (24%) of the population have hypertension and of these only 20% to 30% have controlled blood pressure (BP) (Public Health Agency of Sweden, 2021), which mirrors the situation worldwide. The diagnosis of hypertension means treatment for the rest of a person's life with the aim of achieving target BP of preferably 130/80 mmHg to avoid cardiovascular complications such as stroke or myocardial infarction (Williams et al., 2018). The basis of the treatment is always to modify an unhealthy lifestyle regarding tobacco use, high consumption of alcohol, unhealthy food intake and not being physically active (Williams et al., 2018). That treatment is often combined with medication, mostly with low doses of two or more pharmacological substances to more effectively lower the BP and to avoid side effects (Tsioufis and Thomopoulos, 2017).

Non-adherence to treatment, both pharmacological and non-pharmacological, is the most important factor for uncontrolled BP (Dusing, 2006). According to WHO (Sabate, 2003), patients take only about 50% to 70% of their prescribed doses of antihypertensive drugs. Reported examples of barriers to drug adherence are side effects, the complexity of drug regimens and the impact of medication on daily life (AlGhurair et al., 2012). Barriers to performing lifestyle changes could be an underestimation of one's own cardiovascular risk (Vörös et al., 2018). Several interventions have been tried out to help people to adhere to medication by increasing patients’ knowledge (Gwadry-Sridhar et al., 2013), home BP monitoring (Fletcher et al., 2015; Stergiou et al., 2014), education on hypertension and advice on lifestyle (Cheema et al., 2014), e-counselling programs (Nolan et al., 2014), other digital interventions (McLean et al., 2016) and linking adherence behaviour with habits, giving adherence feedback to patients, using dose dispenser or other special packaging and motivational interviewing (Conn et al., 2015). Interventions to help people to adhere to lifestyle changes mostly concern participating in group treatment (Gomez-Pardo et al., 2016) and counselling using motivational interviewing (Rubak et al., 2005). Some of the different interventions have been shown to be efficient to some extent, but no special intervention has been assessed to be efficient enough to be recommended for general use in health care to help people with hypertension to achieve target BP. Moreover, people with hypertension are reported to have lower quality of life (Korhonen et al., 2011; Trevisol et al., 2011).

There are few studies focused on studying the patients’ experiences of having hypertension. One interview study reported that some patients did not care, some were serious, some adjusted well and some felt frustrated about their hypertension and treatment (Lahdenperä and Kyngäs, 2001). In another study medication was more likely to be identified as a mean of treating hypertension rather than attending to changing lifestyle (Kjellgren et al., 1997). With this limited knowledge of patients’ experiences, it is hard to know what interventions would be the most suitable to help them to achieve BP control. Therefore, there is a need for more studies about what problems patients with hypertension are facing, how they manage these challenges, how they feel and what requirements they believe are important to have in their treatment. This knowledge could then be used as a foundation and incentive for using effective interventions for this large group of people with hypertension.

## 2 Aim

To explore people's experiences of having hypertension.

## 3 Methods

### 3.1 Design

This study has an inductive design based on qualitative interviews, with persons diagnosed with hypertension in order to probe for experiences of patients living with hypertension. The analysis was carried out with qualitative content analysis according to Graneheim and Lundman (2004).

## 4 Sample and participants

The inclusion criterion for this study was persons >20 years of age, diagnosed with hypertension at least 6 months previously and able to read and speak Swedish. To ensure that the population is representative of a region in the south of Sweden, participants were included from both cities and from the countryside.

The participants consisted of 12 persons (9 women/3 men), with a median age of 72 years (range 42–80 years). They had their hypertension diagnosis a median 20 years (range 6 month–39 years, 3 missing data).

## 5 Data collection

Letters containing information about the study and a request for permission to contact nurses working with patients with hypertension were sent to 21 heads of health centres. They all gave their consent and provided names and contact details to nurses who led a hypertension clinic or met many patients with hypertension. Twelve of the nurses recruited 25 patients.

Two university teachers with a master's degree contacted these 25 patients by telephone, and 12 of them agreed to participate. The time and place for the interviews were arranged by telephone and all participants wanted to be interviewed in their home. The interviews were conducted during 2017 and 2018.

During the interviews, following an interview guide, the participants were asked to describe their experiences of living with hypertension. Questions used were e.g. “How do you experience to have elevated blood pressure” and “What do you think about taking medicines to treat your elevated blood pressure?”. Follow-up questions were used to deepen the descriptions of their experiences.

Each interview lasted from 22 to 55 min (median 35 min) and was audio-recorded and transcribed verbatim.

### 5.1 Data analysis

In order to find the subjective meaning in the interview transcripts, qualitative content analysis was performed (Graneheim and Lundman, 2004). All the authors read the transcripts several times to gain an overall understanding and grasp of both the manifest and latent content (Graneheim and Lundman, 2004). Then the meaning was searched for in all parts of the text using the computer program NVivo 12, where meaning units were identified and labelled with a code. The various codes were compared based on their differences and similarities and sorted into nine sub-categories, which then formed three categories. Selected quotations are used to illustrate the findings. The initial analysis was conducted by the first and second author (HR and ED) independently. All authors then met and discussed the results of the analysis until consensus was reached. Several meetings and discussions were held in the whole group during different phases of the study.

## 6 Ethics

The study adheres in its entirety to the Declaration of Helsinki (WMA, 2000). This study is a part of a larger project that was approved by the regional ethical review board in Lund, Sweden (Dnr 2014/330) and is registered at the ClinicalTrial.gov (NCT02682095).

## 7 Results

The analysis of the interviews with the twelve persons with hypertension resulted in three categories: 1) Adaptation, 2) Collaboration with the staff gives security and 3) Anxiety and uncertainty. The categories with their sub-categories can be found in Table 1. The interviewed persons’ gender or age is not given after each quotation in order to preserve their anonymity.

**Table 1:** Overview of the categories and sub-categories of persons’ experiences of having hypertension.

Categories	Sub-categories
Adaptation	Insight into one's own needs
	Acceptance of the diagnosis
	Working with lifestyle
	Playing down the situation
	Absence of symptoms
Collaboration with the staff gives security	Trust in the staff's assessment
	Creating a knowledge base
Anxiety and uncertainty	Difficulties understanding the meaning of the BP value
	On my own
	Disappointment at not reaching the desired BP goal
	Displeasure with the care

BP = blood pressure.

### 7.1 Adaptation

#### 7.1.1 Insight into one's own needs

The persons described how they had personal responsibility for how to manage their hypertension and had become aware of prioritizing their health. They knew about the importance of eating healthy food and being physically active but could have a hard time living up to it. Some felt that they had control by measuring their BP themselves, while others wanted the staff to do it. One opinion was that the staff should be compliant with them so that they had influence over their care and could have support when needed. They said that they had the strength to make healthy lifestyle changes and use new tools such as a dose dispenser or attending a course on how to stop smoking.

And there I think, I would say, the high blood pressure has given me tools to do it because, well, if you think of life and death, because I want to live because I have more to explore in life. But, so, I want to say that you have to look positively at what you can do, but it's surely essential to make your changes. (Interview 10)

#### 7.1.2 Acceptance of the diagnosis

Receiving the hypertension diagnosis was difficult and worrying at the beginning, when acceptance of the diagnosis required hard work. The persons had to find ways to incorporate the new lifestyle into their everyday life and to take their medicine. They expressed thoughts about not being immortal and about hypertension affecting the body, which gave them insight to be careful to take the prescribed medicines. Although the trouble it entailed to adjust the doses, cope with the side effects and take the medicine was a daily reminder of the diagnosis, they expressed gratitude for the medicine and said that they now felt fine.

I have had a life crisis, I have really had a midlife crisis at age 50. Yes, and it's exactly that, the feeling that you're not immortal any more, but you can have […] and kick the …. Already from the age of 50, things happen, things like this with the heart. (Interview 8)

#### 7.1.3 Working with lifestyle

The persons described an almost persistent ongoing struggle to achieve lifestyle changes and maintain the changes once achieved. They were conscious that the changes would be lifelong and they understood which strategies worked and what pitfalls they had as individuals. Sometimes, however, they permitted themselves to yield to a temptation. A motivator to attend to lifestyle changes was the goal of lowering their BP.

You work 10 h and have a half-hour break and that break you have to take in your car. So this thing with the food just completely failed, with the Weight Watchers thinking, and writing down and eating, you see. (Interview 5)

#### 7.1.4 Playing down the situation

Opinions were also expressed by the persons that mirrored a need to play down the fact of having hypertension because it was so common and nothing to be upset or think about. Taking the medicines was perceived to be enough and there was no interest in making any lifestyle changes. Having hypertension was of minor importance and living in the present without any restrictions was central.

No, no, it's high blood pressure, nearly everybody has that. So, it is a little bit like this, that nobody bothers, it's nothing to worry about, no. (Interview 4)

#### 7.1.5 Absence of symptoms

It was revealed that symptoms of hypertension are not easy to recognize, as a result of which the elevated BP was detected only when the persons visited the health centre for some other reason. No differences were perceived after treatment was initiated.

No, if I hadn't gone there, I wouldn't have understood it. That's what's so strange; I have never really, from the blood pressure, felt anything special. (Interview 3)

### 7.2 Collaboration with the staff gives security

#### 7.2.1 Trust in the staff's assessment

The interviewed trusted the staff's assessment that their BP was satisfactory and that the staff told them how often they needed to measure their BP. That could mean that the patient did not need to visit the health centre so often, maybe only once a year. In some cases, the person, in consensus with the physician, moderated the medicines when the trust was mutual. In other cases, the persons just did as the staff told them. Continuity as regards who did the measurement was important. Usually it was performed by the district nurse (a registered nurse specialized in primary care), who then talked to the physician if any problems occurred. The persons’ opinion was that they often had received support and good information from the staff about risks of hypertension, diet and exercise, possible side effects of the medication, keeping a healthy body weight and being aware of their target BP value.

I have the same person all the time that I meet for the blood pressure –, who measures me. So, there, so there is one…. (Interview 10)

#### 7.2.2 Creating a knowledge base

The persons created their own kind of knowledge, such as eating healthy food in general with reduced levels of sugar, salt, or even following a LCHF diet and eating smaller portions. A knowledge base was built through thinking on one's own and receiving information from the staff, friends, the Internet and books. Gaining a knowledge base gave a feeling of safety. Knowing that the BP was at a good level through treatment, with both medication and lifestyle changes increased that feeling. They also felt satisfied engaging in a certain amount of physical activity e.g. dancing once a week and eating food that they felt was good. One person experienced that it even was possible to control the BP through deep breathing. Persons also deviated from the prescription of drugs by the physician based on how they felt, occurrence of side effects and on special occasions where they believed they should alter their medication, e.g. taking no tablets the day before they were scheduled to measure their BP.

I know that it helps if one goes for a walk and if you eat a lot of vegetables, it's good, that much I know. (Interview 1)

### 7.3 Anxiety and uncertainty

#### 7.3.1 Difficulties understanding the meaning of the blood pressure value

The interviewees found it hard to understand why it was so difficult for them to reach their target BP. The meaning of high BP was a blur for the persons. Although measuring the BP was stressful, they measured it now and then, sometimes also after having coffee or a cigar, and at the same time they did not always trust their own measurement. They wondered about why the value sometimes was high and sometimes lower after only five minutes. Questions emerged about whether they had the right kind of medicine but some were at the same time reluctant to change it again or to take medicines at all. Having haemorrhoids, tremor tendency or nosebleeds were connected to hypertension and when any symptom, e.g. dizziness, occurred, they measured the BP themselves or went to the health centre to have it measured.

Yes, and then you can think that, if the blood pressure is high all the time then you can go on having dizziness and all sorts of things, but I must say that I have tried to be observant all the time. As soon as I have felt anything, I have thought about the pressure first, before anything else. Yes, it is there [an alarm all the time], it has become a thing that you, both that you worry of course and… (Interview 3)

#### 7.3.2 On my own

It was revealed that the persons not only felt but also were lonely in their process of both accepting and managing their hypertension and some had very little knowledge about hypertension and its treatment. Sometimes the persons did not want to tell other people that they had hypertension. It was a kind of shame in that they perhaps did not make enough lifestyle changes to manage their BP. There was even a feeling that they might have induced the hypertension by letting themselves e.g. become overweight. They also made their own decisions about stopping the medication due to side effects, although the staff wanted them to be patient as the side effects were expected to disappear over time.

First I got, what was it now, Enalapril, from which I felt really bad so I stopped taking it. I didn't care, I felt sick and got headache from it and I had never had that before, also strange […] Yeah, I thought that it would get better, I only took the tablets for a week […] Well, they wanted me to take them longer, so that my body could get used to it, but I thought I didn't want to go around feeling that bad. No, so I stopped again […] Well, and then I felt fine again. (Interview 7)

#### 7.3.3 Disappointment at not reaching the desired blood pressure goal

Although the persons with hypertension had implemented different lifestyle changes they expressed disappointment that the BP was not affected as they had expected, despite having put a great effort into losing weight, for example. Moreover, anxiety was induced when they were asked to come back for another BP measurement, as the reading for the day was too high.

[You were pretty high there, at 150] At that time, I just got a new appointment for a new measurement. So I went home and was disappointed, thinking “Oh no, what is it now?” (Interview 2)

#### 7.3.4 Displeasure with the care

The persons expressed disappointment in those cases where the staff did not catch their concerns and uncertainty early in the process. When persons were not notified for follow-up visits as agreed they developed a feeling of mistrust. The persons perceived it as offhand behaviour and felt alone in their situation. Mistrust also arose when the BP was measured incorrectly (e.g. there was no time for the person to rest before measurement) or when an inaudible BP was not rechecked.

Persons expressed displeasure when they did not receive information about complications of hypertension, why a 24-hour measurement was needed to fully diagnose hypertension, or recommendations about how their diet could be changed in order to improve their situation. The response from the staff was experienced as impersonal when only standard questions were asked, when different messages were given by different professionals, and when there was a lack of continuity, follow-up and collaboration within the care team. In order to participate in an optimal way and get support from the care team it was felt that it was necessary that the patient had the ability, insight and power to demand this.

So it's the health care, or the treatment, or how you are taken care of, so if you go there often it is a nurse aide or some assistant who takes the blood pressure. So if it is too high, she goes bananas and “We must let the doctor have a look at this.” And so on, and so on, but then not much more happens, that is probably my reaction. (Interview 9)

## 8 Discussion

The analysis of the interviews revealed that the persons with hypertension felt that it was their own responsibility how they managed their hypertension and they found ways to accept and adapt to the diagnosis. The persons continuously attended to necessary lifestyle changes, at the same time there were expressions of a need to play down the seriousness of having hypertension, which was strengthened by the fact that they did not feel any differences after treatment started. The persons trusted the staff to assess their BP values and felt satisfied in creating their own knowledge base from different sources. On the other hand, there were also expressions of concern or anxiety as a result of displeasure with the care received and difficulties in fully understanding the meaning of fluctuating BP values. Moreover, the persons felt lonely as it was not easy to tell other people about their diagnosis. Having hypertension was sometimes perceived as shameful because their lifestyle may have had an impact on it and they did not always have the strength to make the desired lifestyle changes to achieve their BP goal.

The persons in our study reported that their hypertension most often was detected when they sought care for some other ailment. That this is the state of things was also found almost 20 years ago (Kjellgren et al., 1997). Getting the diagnosis was a shock, as expressed by our informants, which for some induced a need to play down the situation. This might be explained by the fact that there are no easily identified symptoms for persons to suspect, on their own, that they might have elevated BP or motivate them to change their lifestyle and take medicines. It is reported (Kjellgren et al., 1998) that persons after treatment felt fewer symptoms such as headache, dizziness, palpitation of the heart and tiredness than before treatment. The trouble is that a number of diseases or stress in a person's everyday life can cause such symptoms.

The importance of having good cooperation and trust between health care personnel and persons with hypertension was revealed in our study as *Cooperation with staff gives security*. Being taken seriously and listened to in a trustful partnership as in person-centred care (Ekman et al., 2011) may be pivotal for the persons to develop a deeper understanding of how to manage their self-care. Managing self-care for persons with hypertension means performing necessary lifestyle changes and most often taking medicines. Continuity is also an important part of building trust (Kristjansson et al., 2013). The persons in our study felt secure knowing that they would be well treated by “their” doctor or nurse, when they contacted their health centre for a consultation. Knowing and feeling trust in one's nurse could form the basis for counselling where a person could feel safe in talking about what conceptions and preferences he or she has about e.g. diet, exercise and food. In our study, the interviewees revealed that they built their own knowledge bank with information from friends, neighbours, staff and the Internet. Some of this accumulated knowledge was sometimes incorrect, which could be of interest for the nurse to get to know in order to help the persons to have a correct foundation for making decisions about their treatment. If the persons make a positive decision, i.e. engage in and adhere to the mutually decided treatment, the possibility to achieve controlled BP increases. Persons with controlled BP experience higher quality of life (Lee et al., 2020), which it is important for both health care personnel and the affected persons to know.

One way to get a sense of being master of one's problem and getting a greater understanding about the meaning of BP values could be to measure BP at home by oneself after being instructed on how to do so, as recommended in guidelines for home BP measurement (Parati et al., 2021). The benefits are e.g. that it is cost-effective, adherence to treatment increases and the self-measurement gives the persons a tool to get feedback on what it means for the BP to take a drug holiday or half of the prescribed dose of medication. When measuring the BP themselves the persons can also be a valuable partner in reporting the BP values to their physician after changed medication. It is also reported that medication adjustments from an online intervention with home BP measurement gave a mean difference between intervention and control group of −3.4/−0.5 mmHg after one year (McManus et al., 2021). The benefit of participation in group activities to counteract the feeling of being alone in one's situation, and of getting support and knowledge, is well documented (Odgers-Jewell et al., 2017) and is cost-effective (Mash et al., 2015).

In our results, the persons had a hard time telling other people about their diagnosis because of shame about the feeling that their unhealthy lifestyle might have an effect on their elevated BP. These feelings of stigma and being on their own could be eased by empathetic counselling by a nurse trained in motivational interviewing (MI) (Miller and Rollnick, 2013). This counselling should be aimed at normalizing such feelings, trying to look forward instead of back, and helping the persons to identify personal goals for the future regarding their lifestyle. In that way the persons’ self-efficacy (Bandura, 1977) might be strengthened and this is important as without strong self-efficacy, i.e. beliefs in one's own capability to execute certain actions, there will be no changed lifestyle (Gleit, 1992).

Other interventions that can be used in primary care to help persons with hypertension to change lifestyle are e.g. a programme with a peer group strategy, like that studied by Gomez-Pardo et al. (2016), who found significantly improved healthy behaviour regarding exercise, weight, food and tobacco use after one year in the intervention group compared to a control group. Another example is daily use of a mobile-phone-based self-control system, which showed sharply decreased BP over the course of eight weeks (Bengtsson et al., 2016). The persons who benefited the most from the intervention were those who had moderately to highly elevated BP at the start of the study. From a review, Etminani et al. (2020) conclude that there is a need to design multifaceted digital interventions that can be adapted to the patients’ behaviour and include support from peers or family members. The value of nurses and other health care personnel being person-centred in treating person who need to change their lifestyle is highlighted earlier in the text. This can be achieved by training in MI (Miller and Rollnick, 2013), which is a directive, person-centred and efficient counselling technique where the counselled persons find their own way to manage their different challenges (Drevenhorn et al., 2006). To meet persons’ displeasure with care, the nurse and other health care personnel also need guidelines to follow and a supportive organization that encourages further education and starting nurse-led hypertension clinics (Drevenhorn et al., 2007). The nurses would also be strengthened in their profession and clinical work by having a theory directed to hypertension care to lean on and also for further development of the care (Drevenhorn, 2018). Any new intervention implemented in primary care should be followed up and evaluated to promote further development.

To increase credibility (Lincoln and Guba, 1985) and to enrich the variation in the phenomenon, participants were included from both cities and from the countryside in a region in the south of Sweden. There was also variation in how long they had their hypertension diagnosis. However, there were only 12 participants, who could be considered a small number, but despite many reminders it was difficult to find people who were able to participate, and we considered that the interviews were sufficiently rich and varied in content. In spite of this, due to the low number of participants it is not possible to generalize the result. Other limitations are that we do not have information about the participants’ ethnicity and also that we, for those persons who did not want to be interviewed when called, do not know the reasons for making that choice. Two researchers performed the interviews, and they had no connection to the participants regarding their treatment of hypertension. To ensure the dependability of the results, the conducting of the interviews was discussed several times between the authors. The first and second author performed the initial analysis independently. Then all the authors met and the results of the analysis were discussed until consensus was reached (Graneheim and Lundman, 2004). Several meetings and discussions were held during various phases of the study. In addition, the results are illustrated with quotations to further strengthen the credibility of the study (Lincoln and Guba, 1985).

## 9 Conclusion

To meet the needs of persons with hypertension, strategies such as person-centred counselling and care, using digital interventions, following national guidelines and starting nurse-led clinics, may be of help. These strategies can give a foundation for increased self-efficacy, which is crucial for persons to be able to change lifestyle and adhere to prescribed medication in order to achieve BP control.

## Funding

No external funding.

## Contribution of the paper

What is already known about the topic?

Non-adherence to treatment by persons with hypertension gives that only 20–30% of them achieve controlled blood pressure of ≤140/90 mmHg.

Though many interventions have been tried out, no special has been assessed to be efficient enough to be recommended for general use in health care.

What this paper adds

Persons with hypertension reported that they adapted to their diagnosis in different ways, collaboration with the staff gave security, but they still perceived anxiety and uncertainty.

To meet the needs of people with hypertension, strategies such as person-centred counselling and care, using digital interventions, following national guidelines and starting nurse-led clinics, may be of help.

## Declaration of Competing Interest

The authors declare that they have no known competing financial interests or personal relationships that could have appeared to influence the work reported in this paper.
